# *Penthorum chinense* Pursh improves type 2 diabetes mellitus via modulating gut microbiota in *db/db* mice

**DOI:** 10.1186/s12906-023-04136-z

**Published:** 2023-09-09

**Authors:** Jilei Hu, Huibo Xie, Ning Lin, Yan Yang

**Affiliations:** 1 Clinical Nutrition, The General Hospital of Western Theater Command, Chengdu, 610083 P. R. China; 2 School of Public Health, Southwest Medical University, Luzhou, 646000 P. R. China; 3 Environmental health effects and risk assessment Key Laboratory of Luzhou, School of Public Health, Southwest Medical University, Luzhou, 646000 P. R. China

**Keywords:** *Penthorum chinense* Pursh, T2DM, Insulin resistance, Inflammation, Gut microbiota

## Abstract

*Penthorum chinense* Pursh (*P. chinense*) has been traditionally used as hepatoprotective food and medicine for hundreds of years due to its significant antioxidant and anti-inflammatory activities. However, the efficacy and mechanisms of action of *P. chinense* in type 2 diabetes mellitus were not fully understood. In this study, we found that *P. chinense* extract (PCP) supplementation resulted in reduced body weight and hyperglycemia, improved pancreatic tissue injury and insulin sensitivity, and decreased inflammatory cytokines expression in spontaneously diabetic *db/db* mice. 16S rRNA gene sequencing of fecal samples showed that PCP administration decreased the abundance of *Firmicutes* and increased the proportion of *Bacteroidetes* at the phylum level. Moreover, *Muribaculum*, *Barnesiella*, *Prevotella*, and *Mucinivorans* were enriched, with *Desulfovibrio* and *Lactobacillus* lowered at the genus level in *db/db* mice with PCP supplementation. These results suggested that PCP may ameliorate hyperglycemia, insulin resistance, and inflammation by remodeling the gut microbiota in *db/db* mice.

## Introduction

The latest data published by the International Diabetes Federation in 2023 shows that there are approximately 537 million people worldwide suffering from diabetes, with type 2 diabetes (T2DM) accounting for over 90% of these cases. T2DM is a chronic metabolic disease manifested as hyperglycemia with insulin resistance (IR) or relatively inadequate insulin secretion [1]. However, the underlying mechanism and effective prevention/treatment for T2DM are still not fully discovered. As a progressive disease, systemic inflammation and metabolic disruption contribute to various complications caused by abnormal blood glucose. Hence, it is critical to maintain glucose homeostasis in individuals with T2DM.

The gut has been recognized as the biggest “immune organ” for years, and the intestine microbiome living inside was altered by disease, drugs, diet, and environment [2]. The potential role of gut microbiota exhibited in the pathogenesis and progression of T2DM has been reported in both animal and human studies [3, 4]. Significant changes in the structure and composition of the intestinal flora of patients with T2DM, characterized by a decrease in the abundance of probiotic bacteria, such as *Bifidobacterium* and *Roseburia*, and an increase in the abundance of opportunistic pathogens, including *Escherichia coli* and *Desulfovibrio* [5]. The gut microbiota can produce short-chain fatty acids (SCFA), lipopolysaccharides (LPS), secondary bile acids (BA), peptidoglycan, and a variety of other beneficial or harmful metabolites, which in turn act on the host. SCFA can regulate the gut-liver axis to maintain glucose homeostasis and reduce IR, inhibit inflammation, and repair the intestinal barrier [6, 7]. LPS promotes metabolic endotoxemia and low-grade inflammation, which exacerbate IR and T2DM [8]. BA activates receptors including farnesoid X receptor (FXR) and G protein-coupled bile acid receptor 5 (TGR5) to trigger downstream cascades to regulate glucose metabolism [9–11]. Modulation of gut microbiota is increasingly believed as a promising approach for the treatment of T2DM.

*Penthorum chinense* Pursh (*P. chinense*) has been used as a medicinal and dietary herb in China for hundreds of years. It was demonstrated by infrared spectroscopy that polyphenols and flavonoids (kaempferol, quercetin, and pinocembrin-7-O-beta-D-glucoside) were the most abundant biomolecules in *P. chinense* extract (PCP) [12–14]. Notably, polyphenol-rich herbal medicine and functional food like green tea, Pueraria lobata and Moringa oleifera have long been known to offer diabetes-protective properties [15–17]. Previous study had reported that PCP activated the Nrf2 antioxidant pathway by binding to Keap1 protein, and significantly reduced the vascular inflammation induced by high glucose levels [18]. Huang et al. [19] found that polyphenols of PCP possessed a modest hypoglycemic effect by increasing insulin secretion on diabetic rats induced by high-fat diet (HFD) combine with streptozotocin (STZ). Meanwhile, PCP had been reported to inhibit intestinal inflammation in animals, enhance the α diversity of the gut microbiota, and enrich SCFA-producing bacteria [20–22]. However, there is no report about the hypoglycemic effects of PCP in *db/db* mice, and the underlying hypoglycemic mechanism of PCP is still unclear. In addition, the role of gut microbiota in the therapeutic efficacy of PCP in T2DM remains to be further investigated.

The present study attempted to investigate the role of gut microbiota in PCP protecting *db/db* mice from T2DM. We have examined glucose- and lipid-related parameters, inflammatory cytokines, and pathological changes in the pancreas to determine the hypoglycemic effect of PCP. Changes in gut microbiota were analyzed by 16S rRNA sequencing to further identify the relationship among PCP, gut microbiota, and T2DM. The current study may provide a new perspective on the mechanism of PCP in the treatment of T2DM.

## Materials and methods

### Herbal materials and Preparation

The dried *P. chinense* was obtained from Gulin Hongan Pharmaceutical Co., Ltd. (Luzhou, China). Samples were ground into powder and adequately immersed in ultrapure water. Then, the mixture was exhaustively extracted with boiling water three times for 1 h each. Finally, the resulting decoction was concentrated to 1 g/mL.

### Animals experiment

All animal experiments were approved by the Ethics Committee of Southwest Medical University (No.201910-236). The male *db/db* and *db/m* mice (8 weeks) were provided by Changzhou Cavens Co., Ltd. (Changzhou, China). All animals were fed in the SPF Animal Laboratory Center of Southwest Medical University at 12 h light/dark cycle, (23 ± 2) ℃, and (60 ± 5) % relative humidity. All mice accessed food and water freely. After seven days of acclimatization, the fasting blood glucose (FBG) was monitored by a glucometer and glucose test strips (Roche Co., Germany). The *db/db* mice detected with FBG over 11.1 mmol/L were defined as diabetic mice. Eight *db/m* mice were involved in the normal control group (NC) and twenty-four *db/db* mice were randomly divided into three groups: diabetic model control group (DC), metformin group (Met), and PCP treatment group (PCP). The Met group received metformin 150 mg/kg/d (Glucophage, manufactured by Sino-American Shanghai Squibb Pharmaceuticals Ltd.). The PCP group was administered a dose of 1200 mg/kg/d, which was determined according to our earlier pre-experimental results. Allometric scaling to a person with a body weight of 60 kg, the dose corresponds to 8-gram PCP daily, at the tolerable upper intake levels of humans. Only ultrapure water was given to the animals in the NC and DC. All treatments were administered by gavage for four weeks.

### Oral glucose tolerance test

All animals were given 50% glucose solution at a dose of 2 g/kg when OGTT was conducted, and tail venous blood was collected at 0, 30th, 60th, and 120th minutes after oral gavage to measure blood glucose concentration.

### Biochemical analyses

Mice were terminally anesthetized with 1% sodium pentobarbital solution (1 mL/100 g) and collected blood samples and viscera. 1 mL of blood samples was centrifuged at 3500 rpm for 10 min to obtain serum samples, and 0.5 mL of whole blood was centrifuged at 10,000 rpm for 10 min to collect hemocytes for the measurement of glycated hemoglobin A1c (HbA1c). Serum triglyceride (TG), total cholesterol (TC), low-density lipoprotein cholesterin (LDL-C), and high-density lipoprotein cholesterin (HDL-C) were determined by commercial kits (Jiancheng Co., Ltd., Nanjing, China). Fasting serum insulin (FINS), HbA1c, interleukin-6 (IL-6) and tumor necrosis factor-α (TNF-α) were detected by ELISA kits (Meilian Co., Ltd., Shanghai, China). IR is indicated by homeostasis model assessment of insulin resistance (HOMA-IR) with a calculation formula of FBG (mmol/L) × FINS (mU/L)/22.5.

### Pancreas tissue histopathological analysis

The pancreas tissue was prefixed with 2.5% glutaraldehyde solution and fixed with 1% osmium tetroxide solution. The tissue was dehydrated with acetones by gradient, permeated, embedded, cut into approximately 50 nm-thick lice, and stained with uranyl acetate and lead citrate solution. The pancreas slices were observed under transmission electron microscopy (TEM, JEM-1400PLUS, JEOL, Japan).

### Gut microbiota analysis

Randomly selected five mice from each group to collect fecal samples at the end of the intervention. Straightway froze the samples in liquid nitrogen before being preserved at -80 ℃. Fecal bacteria DNA was extracted by the DP4015 fecal genomic DNA extraction kits (Pacific Biosciences Co., California, USA). The purity and concentration of the extracted fecal were measured, and the Illumina MiSeq third-generation sequencing was provided by Wuhan Hope Group Biotechnology Co., Ltd. The raw data was mass filtered and connected with double-ended sequences, and the high-quality sequence was screened. The clustering and annotation of OTU were performed to analyze the diversity index, the community structure, and species abundance at each taxonomic level.

### Statistical analysis

All data were analyzed by SPSS 22.0 statistical software and the results are presented as the mean ± standard deviation (SD). One-way ANOVA analysis was performed to compare the mean differences among multiple groups. In pairwise comparison, the LSD test was used for those with homogeneous variances, and the Dunnett T3 test was used for those with uneven variances. The correlation between the gut microbiota and physicochemical indicators was calculated by Spearman correlation analysis. A *P*-value of less than 0.05 was deemed statistically significant.

## Results

### PCP inhibited the increase in body and organ weights in *db/db* mice

**Fig. 1:**
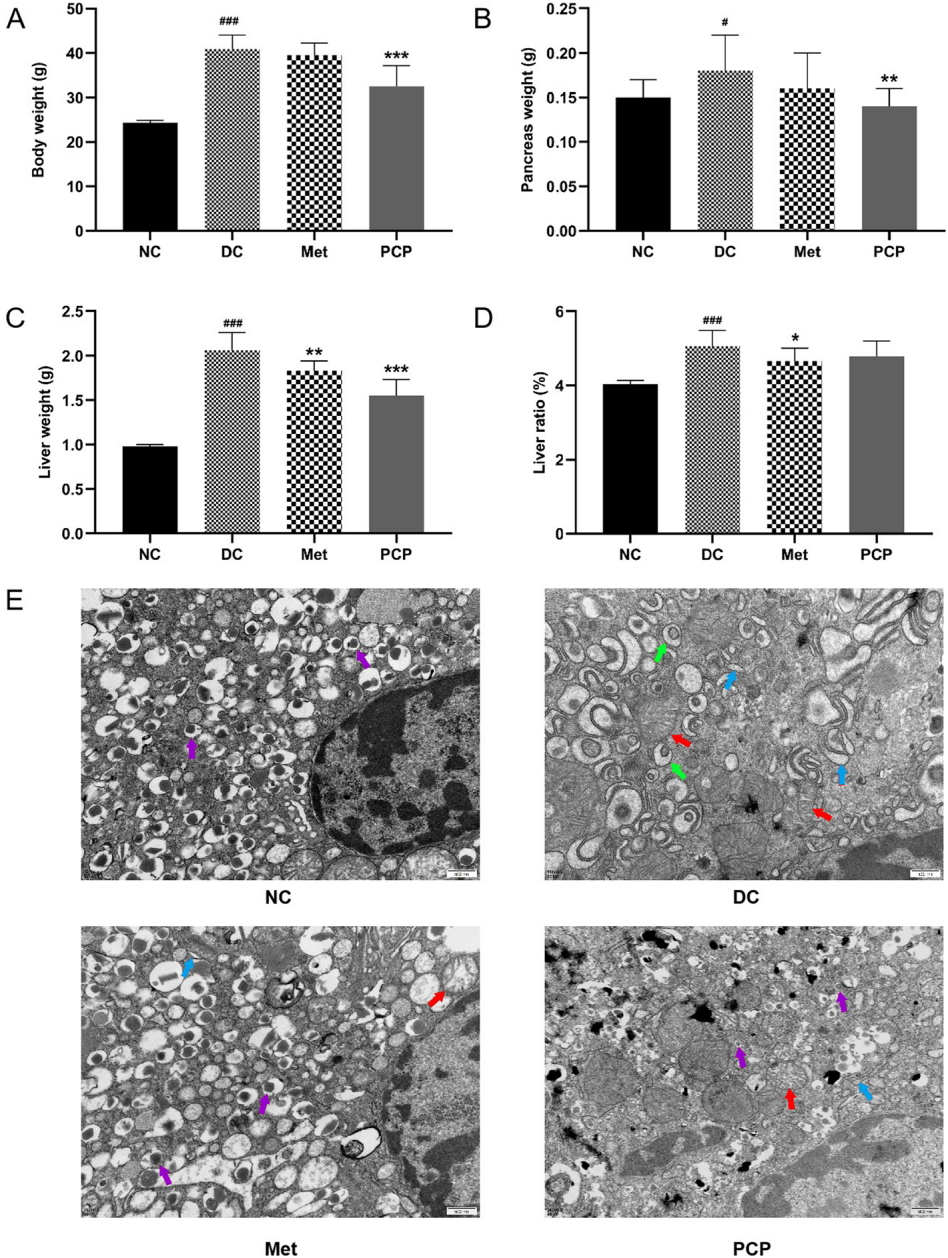
Effect of PCP on body and organ weights in *db/db* mice. (**A**) Body weight, (**B**) Pancreas weight, (**C**) Liver weight, (**D**) Liver ratio and (**E**) representative photomicrographs of TEM observation of pancreatic ultrastructure (scale bar, 500 nm). The purple arrows (**↑**) pointed to pancreatic β cells with abundant β granules, the green arrows (**↑**) pointed to pancreatic α cells with abundant α granules, the red arrows (**↑**) displayed the location of mitochondrial swelling, and the blue arrows (**↑**) displayed the location of endoplasmic reticulum expansion. All data are presented as the mean ± SD (*n* = 8). ^#^, ^##^, and ^###^ indicate *P* < 0.05, *P* < 0.01, and *P* < 0.001, respectively, in the comparison between the NC and DC groups. ^*^, ^**^, and ^***^indicate *P* < 0.05, *P* < 0.01, and *P* < 0.001, respectively, in comparisons between the DC group and the treatment groups

As shown in Fig. 1, the body, pancreas and liver weights as well as liver ratio of *db/db* mice were significantly higher than those of *db/m* mice (*P* < 0.05, *P* < 0.001). After 4 weeks of Met treatment, a significant decrease of the liver and liver ratio, was observed in Met group (*P* < 0.05, *P* < 0.01). After 4 weeks of PCP administration, the body weight, liver, and pancreas weights of PCP group were significantly reduced (*P* < 0.01, *P* < 0.001).

The ultrastructure of the pancreatic tissue was observed by TEM (Fig. 1E), which revealed intact nuclear membranes, a large number of β granules, and well-defined organelles including mitochondria, rough endoplasmic reticulum and ribosomes in islet β cells of *db/m* mice. There was a decrease in β granules among islet β cells of the DC group when compared to the NC group, while the cytoplasm contained large amounts of α particles with some mitochondria slightly swollen, and most of the rough endoplasmic reticulum dilated in a cystic shape. The β granules in islet β cells were augmented in the Met and PCP groups in comparison to that of the DC group, with comparable improvements in both groups. After treatment with Met and PCP, rough endoplasmic reticulum expansion in islet β cells of *db/db* mice was significantly improved and mitochondrial swelling was amended to varying degrees.

### PCP improved FBG, FINS and glucose tolerance in *db/db* mice

**Fig. 2:**
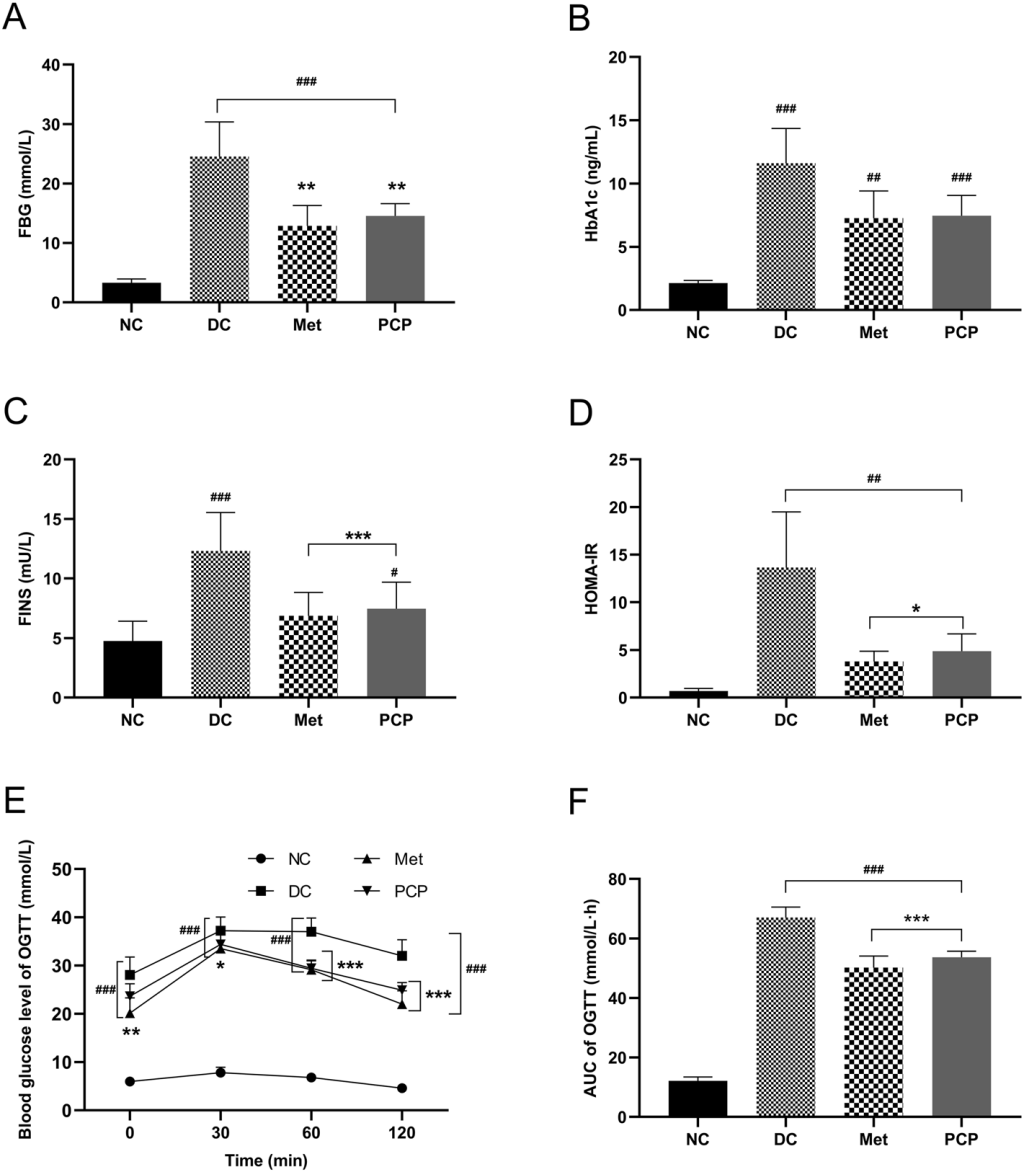
Effect of PCP on blood glucose, insulin, and glucose tolerance in *db/db* mice. (**A**) FBG, (**B**) HbA1c, (**C**) FINS, (**D**) HOMA-IR, (**E**) The curve of OGTT and (**F**) AUC of OGTT. All data are presented as the mean ± SD (*n* = 8). ^#^, ^##^, and ^###^ indicate *P* < 0.05, *P* < 0.01, and *P* < 0.001, respectively, in the comparison between the NC and DC groups. ^*^, ^**^, and ^***^indicate *P* < 0.05, *P* < 0.01, and *P* < 0.001, respectively, in comparisons between the DC group and the treatment groups

As described in Fig. 2A-D, *db/db* mice exhibited significant hyperglycemia and hyperinsulinemia compared to *db/m* mice (*P* < 0.001). FBG in *db/db* mice was reduced to varying degrees after treatment with metformin and PCP (*P* < 0.001). The levels of HbA1c in *db/db* mice were lowered by 37.41% and 35.69% respectively, but without significant differences compared to the DC group (*P* > 0.05). FINS and HOMA-IR were significantly lower in *db/db* mice after administration of metformin and PCP (*P* < 0.001, *P* < 0.05).

The results of OGTT were shown in Fig. 2E-F. The blood glucose values and AUC of the *db/db* mice were significantly higher than those of the *db/m* mice at all time points (*P* < 0.001). After four weeks of treatment with metformin, the blood glucose values and AUC of the Met group were significantly lower than those of the DC group at all time points (*P* < 0.05, *P* < 0.01, *P* < 0.001). Blood glucose values at 60th and 120th minutes and AUC of the PCP group were significantly lower than those of the DC group (*P* < 0.001).

### PCP decreased blood lipid levels in *db/db* mice

**Fig. 3:**
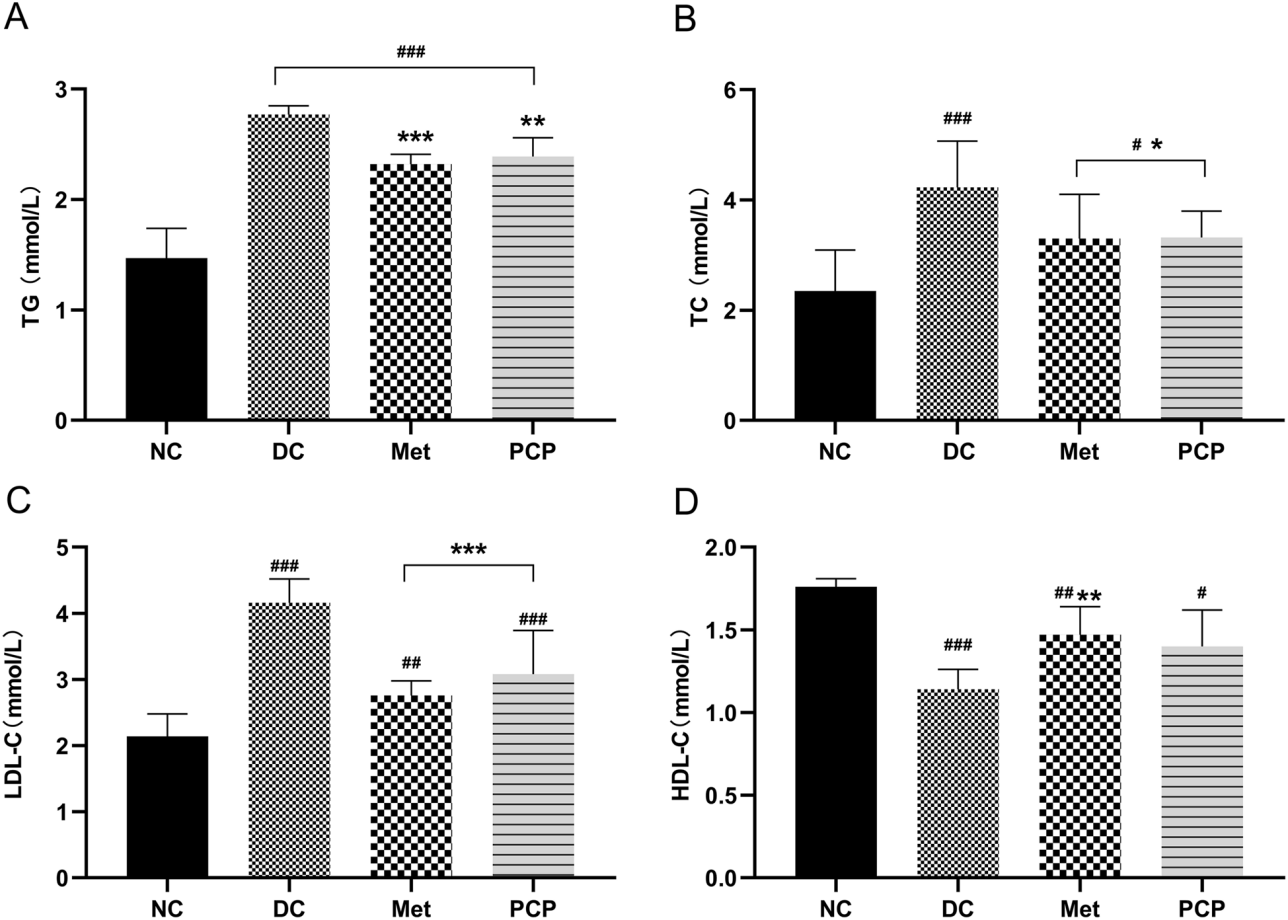
Effect of PCP on blood lipid in *db/db* mice. (**A**) TG, (**B**) TC, (**C**) LDL-C, and (**D**) HDL-C. All data are presented as the mean ± SD (*n* = 8). ^#^, ^##^, and ^###^ indicate *P* < 0.05, *P* < 0.01, and *P* < 0.001, respectively, in the comparison between the NC and DC groups. ^*^, ^**^, and ^***^indicate *P* < 0.05, *P* < 0.01, and *P* < 0.001, respectively, in comparisons between the DC group and the treatment groups

Serum TC, TG, and LDL-C levels were significantly elevated and HDL-C levels were significantly depressed in *db/db* mice when compared to *db/m* mice (Fig. 3, P < 0.001). After treatment with Met and PCP, the levels of TC, TG, and LDL-C in *db/db* mice were significantly decreased (*P* < 0.05, *P* < 0.01, *P* < 0.001). The levels of HDL-C in the Met group were effectively increased (*P* < 0.01), and those in the PCP group were raised to some extent but the difference was not statistically significant (*P* > 0.05).

### PCP attenuated inflammatory cytokine expression in *db/db* mice

**Fig. 4:**
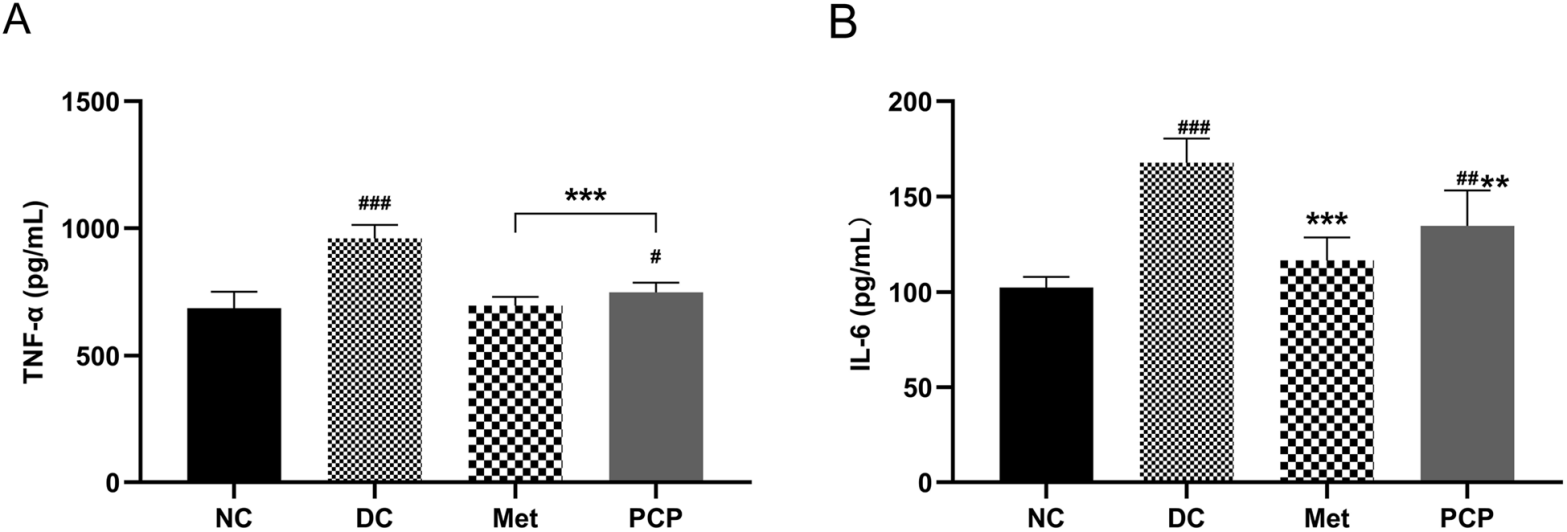
Effects of PCP on inflammatory cytokine in *db/db* mice. (**A**) TNF-α and (**B**) IL-6. All data are presented as the mean ± SD (*n* = 8). ^#^, ^##^, and ^###^ indicate *P* < 0.05, *P* < 0.01, and *P* < 0.001, respectively, in the comparison between the NC and DC groups. ^*^, ^**^, and ^***^indicate *P* < 0.05, *P* < 0.01, and *P* < 0.001, respectively, in comparisons between the DC group and the treatment groups

As shown in Fig. 4, the contents of TNF-α and IL-6 were significantly higher in the DC group than in the NC group (*P* < 0.001). After treatment with Met and PCP, the expression of TNF-α and IL-6 was significantly decreased in the Met and PCP groups as compared to the DC group (*P* < 0.01, *P* < 0.001).

### PCP modified the diversity and richness of the gut microbiota in *db/db* mice

**Fig. 5:**
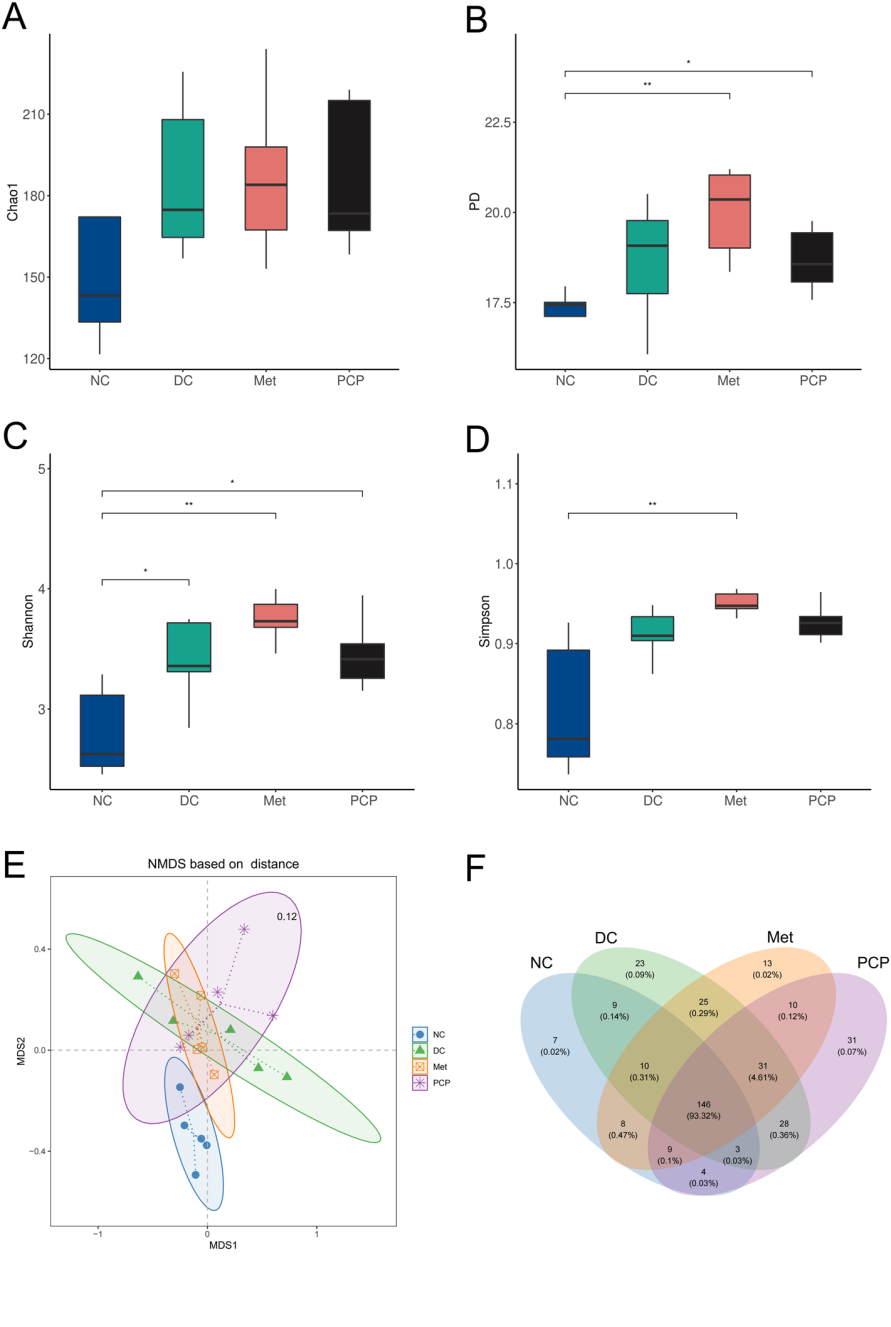
Effects of PCP on the diversity and richness of the gut microbiota. (**A**) Chao1 index, (**B**) PD index, (**C**) Shannon index, (**D**) Simpson, (**E**) NMDS analysis, and (**F**) Venn diagram based on comparisons of the overlapping and exclusive OTUs of the four groups. ^*^, ^**^ and ^***^ indicate *P* < 0.05, *P* < 0.01 and *P* < 0.001, respectively

As shown in Fig. 5A-D, PD and Chao1 index were higher in *db/db* mice than those in *db/m mice*. In particular, PD was significantly raised in the Met and PCP groups than that in the NC group after metformin and PCP intervention (*P* < 0.05). A significantly higher Shannon index was observed in *db/db* mice as compared to *db/m* mice (*P* < 0.05). In parallel, the Simpson index of the Met group was significantly elevated in comparison to the NC group after treatment with metformin (*P* < 0.05).

Non-metric multidimensional scaling (NMDS) analysis showed a stress value of 0.12, where a stress value of less than 0.2 indicates reliable data results. NMDS analysis suggested noticeable differences in the composition of the microbial communities among the four groups. After treatment with PCP, the composition of the gut microbiota was closer to that of the NC group (Fig. 5E). The Venn diagrams showed that 7, 23, 13, and 31 unique OTUs were found in the NC, DC, Met, and PCP groups respectively, and 146 OTUs were identical across the four groups (Fig. 5F).

### PCP altered the composition of the intestinal flora in *db/db* mice

**Fig. 6:**
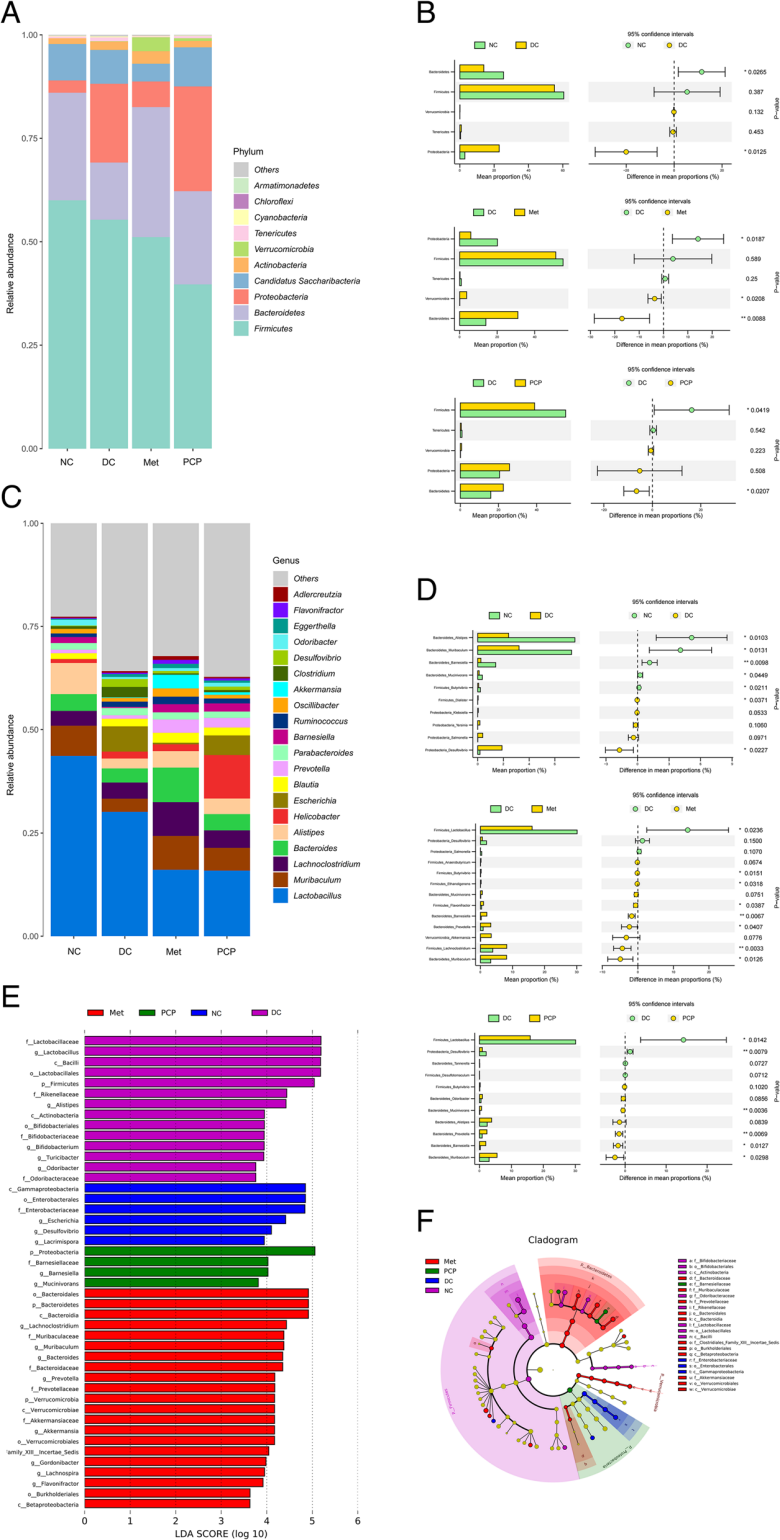
Effects of PCP on the composition of the gut microbiota in *db/db* mice at phylum and genus levels. (**A**) Relative abundance of phylum level. (**B**) Metastats analysis at phylum level among NC, Met, PCP and DC groups. (**C**) Relative abundance of genus level. (**D**) Metastats analysis at genus level among NC, Met, PCP and DC groups. (**E**) Linear discriminant analysis. (**F**) LEfSe analysis. ^*^, ^**^ and ^***^ indicate *P* < 0.05, *P* < 0.01 and *P* < 0.001, respectively

*Firmicutes*, *Bacteroidetes*, *Proteobacteria*, and *Candidatus Saccharibacteria* were the dominant microflora at the phylum level, especially *Firmicutes* and *Bacteroidetes* accounted for over 60% (Fig. 6A). Metastats analysis revealed that the relative abundance of *Bacteroidetes* was significantly decreased while the abundance of *Proteobacteria* was increased in the DC group when compared to the NC group (Fig. 6B, P < 0.05). These trends were effectively reversed by the administration of metformin and PCP. In the Met group, the relative abundance of *Bacteroidetes* and *Verrucomicrobia* increased while that of *Proteobacteria* decreased (*P* < 0.05, *P* < 0.01). Furthermore, the relative abundance of *Bacteroidetes* increased while that of *Firmicutes* decreased in the PCP group (*P* < 0.05).

As shown in Fig. 6C-D, the relative abundance of *Alistipes*, *Muribaculum*, *Barnesiella*, *Mucinivorans*, and *Butyrivibrio* markedly declined in the DC group compared to the NC group, while those of *Desulfovibrio* and *Dialister* significantly elevated at the genus level (*P* < 0.05, *P* < 0.01). In the Met group, the relative abundance of *Muribaculum*, *Lachnoclostridium*, *Prevotella*, *Barnesiella*, *Flavonifractor*, *Ethanoligenens*, and *Butyrivibrio* was significantly higher, while the relative abundance of *Lactobacillus* was significantly lower than those in the DC group (*P* < 0.05, *P* < 0.01). Compared with the DC group, six communities in the PCP group showed significant changes at the genus level, with significantly higher relative abundances of *Muribaculum*, *Barnesiella*, *Prevotella*, *Mucinivorans*, and lower relative abundances of *Desulfovibrio* and *Lactobacillus* (*P* < 0.05, *P* < 0.01). Notably, the relative abundance of *Akkermansia*, *Alistipes*, and *Butyrivibrio* was higher in the PCP group as compared to the DC group, but the difference was not statistically significant.

The dominant bacterial communities in each group were analyzed by linear discriminant analysis effect size (LEfSe), with a total of 45 biomarkers scoring more than 3 in linear discriminant analysis (LDA) (Fig. 6E-F). The superior genera of the NC group were *Lactobacillus, Alistipes*, *Bifidobacterium*, *Turicibacter*, and *Odoribacter.* The DC group was represented by *Lacrimispora*, *Desulfovibrio*, and *Escherichia*. The superior genera of the Met group were *Lachnoclostridium*, *Muribaculum*, *Bacteroides*, *Prevotella*, *Akkermansia*, *Gordonibacter*, *Lachnospira*, and *Flavonifractor*. The representative genera of the PCP group were *Barnesiella* and *Mucinivorans*.

### Correlation between T2DM-related parameters and gut microbiota

**Fig. 7:**
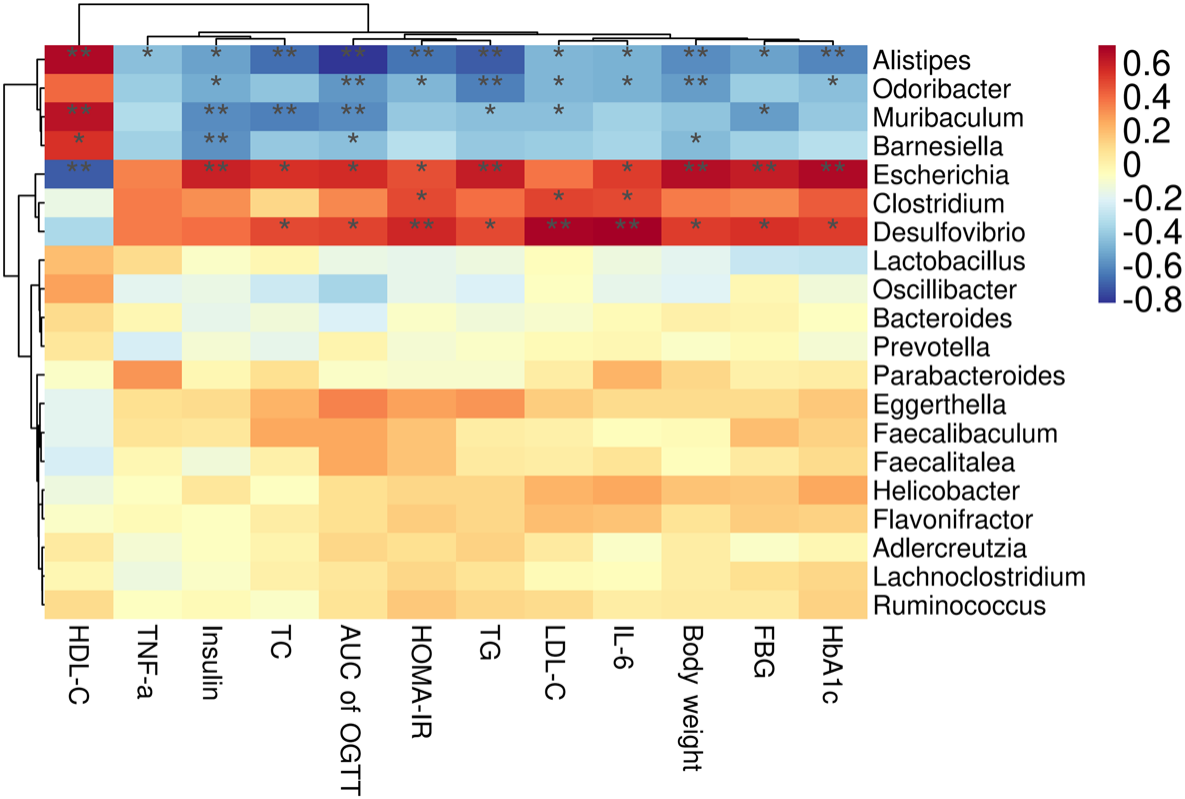
Heatmaps of the correlation between the T2DM-related parameters and the gut microbiota at the genus levels. ^*^, ^**^ and ^***^ indicate *P* < 0.05, *P* < 0.01 and *P* < 0.001, respectively

As demonstrated in Fig. 7, the results of the correlation analysis between the top 20 high abundance genera and the diabetes-related indicators. *Desulfovibrio* and *Escherichia* showed positive correlations with FBG, HbA1C, body weight, TC, TG, LDL_C and IL-6 (*P* < 0.05, *P* < 0.01). *Alistipes*, *Odoribacter*, and *Muribaculum* showed strong positive correlations with the majority of glucose- and lipid-related indicators, inflammatory factors, and body weight were negatively correlated with them (*P* < 0.05, *P* < 0.01).

## Discussion

T2DM is a metabolic disease characterized by chronic hyperglycemia with impaired insulin secretion and/or utilization. We evaluated the effects of PCP on blood glucose and lipids, IR, inflammatory cytokines, pancreatic tissue damage, and gut microbiota in *db/db* mice. It was found that PCP was effective in suppressing hyperglycemia, hyperinsulinemia, and the expression of inflammatory cytokines (IL-6 and TNF-α), and boosting glucose tolerance in *db/db* mice. These health-promoting effects of PCP may be related to the modulation of the structure and composition of the gut microbiota.

Growing evidence shows that dysbiosis of the gut microbiota is an underlying causative agent of T2DM and plays a potential regulatory role in its pathogenesis and progression [23]. The structure and composition of the intestinal flora play an essential role in the host’s health status. Previous studies have reported that PCP can significantly change the composition of the gut microbiota in healthy mice, common carp, and broilers by upregulating the relative abundance of *Bacteroidetes* and decreasing the amount of *Firmicutes*, contributing to a health-promoting intestinal environment [20, 21, 24]. Gut microbial changes characterized by a higher abundance of *Firmicutes* and a lower abundance of *Bacteroidetes* are frequently reported in patients with T2DM [25, 26]. *Firmicutes* are beneficial in stimulating host appetite and energy metabolism, and their abnormal increase may lead to obesity and IR in hosts. *Bacteroidetes* are considered to be the most common beneficial bacteria in the gut. The ratio of *Firmicutes* to *Bacteroidetes* is positively correlated with body weights and negatively associated with glucose tolerance [27]. In this study, PCP decreased the abundance of *Firmicutes* and increased the abundance of *Bacteroidetes* in *db/db* mice as expected, which was consistent with the trend in their body weight, lipids, blood glucose, and glucose tolerance. In addition, we found that PCP significantly raised the relative abundance of *Mucinivorans* at the genus level. It had been reported that *Mucinivorans* not only alleviated obesity and improved insulin sensitivity in hosts, but also regulated lipid metabolism and inhibited cholesterol synthesis [28].

Chronic inflammation induced by high glucose can lead to impaired islet β-cell function and worsen IR [29, 30]. As shown in the results, PCP treatment significantly decreased the expression of IL-6 and TNF-α as well as lowered the levels of FINS and HOMA-IR in *db/db* mice. It was further observed under TEM that PCP significantly improved pancreatic tissue edema and rough endoplasmic reticulum expansion and mitochondrial swelling of β cells, indicating that PCP could attenuate pancreatic injury and enhance insulin sensitivity in *db/db* mice by suppression of inflammation. Previous studies have shown that PCP can suppress inflammation in the body by activating the Nrf2/HO-1, AMPK/p62/Nrf2/mTOR, or SIRT1/AMPK signaling pathways [31–33]. Moreover, the gut microbiota can influence systemic metabolism and inflammatory states through the metabolism of nutrients and the release of anti- and pro-inflammatory factors. LPS is a major component of the cell wall of gram-negative bacteria and activates toll-like receptor 4 on the surface of macrophages to secrete pro-inflammatory cytokines upon entry into the bloodstream, causing endotoxemia [34]. In contrast, SCFA enhances barrier integrity by upregulating tight junction protein expression and increasing transmembrane resistance to reduce LPS entering the circulation and inhibit the expression of inflammatory factors [35]. Furthermore, as one of the most abundant microbial metabolites in the gut, SCFA has been shown to maintain glucose homeostasis and reduce host IR through the gut-pancreas-liver axis [36]. SCFA can increase insulin sensitivity and inhibit hepatic gluconeogenesis by binding to GPR41/43 on hepatic and pancreatic cell membranes to activate AMPK signal and cAMP-dependent protein kinase A leads to the phosphorylation of downstream target proteins [37].

A lack of SCFA-producing bacteria and an increase in LPS-producing bacteria are common manifestations of gut microbiota dysbiosis in patients with T2DM [38, 39]. In the present study, PCP treatment significantly decreased the relative abundance of *Desulfovibrio* and *Lactobacillus*, and increased the relative abundance of *Muribaculum*, *Prevotella*, and *Barnesiella* in *db/db* mice at the genus level. *Desulfovibrio* is the representative LPS-producing bacterium that converts sulfate into hydrogen sulfide, which disrupts the gut barrier and increases gut permeability [40]. *Muribaculum* and *Prevotella* are known to produce butyrate primarily, which is known to inhibit the expression of proinflammatory cytokine genes in adipocytes and contribute to preserving the integrity of the intestinal barrier [41, 42]. In addition, PCP was significantly enriched for the anti-inflammatory bacteria *Barnesiella* and *Muribaculum*. *Barnesiella* is associated with some immunomodulatory factors, such as IL-10, which can suppress inflammation in the gut [43, 44]. Ye’s study found that IL-10-/- mice with higher abundance of *Barnesiella* developed lower levels of colitis disease [45]. Moreover, Gao’ and Tao’ teams had proven that PCP treatment resulted in upregulating the expression of the anti-inflammatory factor IL-10 in the intestine of common carp and serum of dogs [20, 46], but further studies are needed to demonstrate whether it is associated with an increased abundance of *Barnesiella* in the gut. *Muribaculum* is an important mucin monosaccharide forager that inhibits *Clostridium* difficile colonization of the intestine, contributing to the reduction of intestinal inflammation and the maintenance of intestinal microecological stability [47]. In summary, we hypothesized that PCP suppressed intestinal inflammation and maintained intestinal homeostasis by increasing the abundance of SCFAs-producing and anti-inflammatory bacteria while decreasing the abundance of LPS-producing bacteria to ameliorated hyperglycemia and IR in *db/db* mice.

Notably, the relative abundance of *Lactobacillus* showed a significant decrease after PCP treatment, which may be contrary to some studies [48, 49]. Some ideas were that the increase of *Lactobacillus*, such as *Lactobacillus johnsonii* and *Lactobacillus rhamnosus*, can inhibit the growth of pathogens by secreting lactic acid to decrease the pH of the gut, maintain the intestinal barrier and repair the impaired glucose tolerance [50]. However, Karlsson et al. [51] had reported that *Lactobacillus*, classified to the phylum of *Firmicutes*, were notably enriched in T2DM individuals and positively correlated with FBG and HbA1c. Similarly, Li’s team proposed that PCP could alleviate dyslipidemia and IR via gut microbiota-bile acid-axis to reduce the abundance of bile salt hydrolase (BSH)-producing bacteria including *Lactobacillus, Clostridium_IV* and *Clostridium_XIVb* [22]. They also found that PCP treatment markedly decreased the protein levels of FXR in the ileum of HFD-fed mice and then downregulated the expression of fibroblast growth factor 15 (FGF15) due to the increased levels of taurine-conjugated BAs in feces. Taurine-bound muriatic acid present in mice is a natural antagonist of FXR [52]. FXR activated by taurine-conjugated BAs induces expression of FGF15, which promotes the uptake of chylomicrons by adipocytes through activation of the schizogenic-activated protein kinase pathway to promote adipocyte uptake of glucose to improve insulin sensitivity and glucose tolerance [53]. Moreover, He et al. [54] had revealed that *Lactobacillus* was mainly negatively associated with the expression levels of the liver PI3K/Akt/FoxO1 and GPR43/AMPK signaling pathways by correlation analysis. The PI3K/Akt/FoxO1 signaling cascade has been recognized as the principal mechanism of insulin signal transduction that plays a critical role in the modulation of hepatic IR [55]. Therefore, we conjectured whether the improvement of glycemia and IR in *db/db* mice by PCP was also associated with the gut-pancreas-liver axis. However, there was insufficient evidence for this assumption and further exploration will be needed.

The increased diversity of intestinal flora is beneficial for maintaining the stability of gut function [56]. Based on the results of α diversity analysis, we found that Chao1, PD, Simpson, and Shannon indexes were increased to some extent in *db/db* mice after PCP treatment. Interestingly, the PD and Shannon indexes were significantly lower in the NC group than those in the DC group. This was contrary to the results of previous studies, which overwhelmingly concluded that individuals with T2DM should have lower intestinal flora diversity than normal individuals [57]. Further analysis revealed that the results of Sun’s study were in agreement with ours, that *db/db* mice had higher α diversity of gut microbiota than *db/m* mice, and the experimental animals for both studies were from the same institution [58]. Therefore, we hypothesized that the results above may be related to the breeding environment and individual differences of animals.

To the best of our knowledge, this is the first time to investigate the effects of PCP on gut microbiota in *db/db* mice. Our findings provided a new perspective on the possible mechanism of PCP application in diabetes. However, there are some limitations to our study. Firstly, PCP, as a new novel food, was only approved to be consumed in the form of tea bags in China. Therefore, *db/db* mice were selected to be intervened with an aqueous extract of *P. chinense* to simulate the way of human consumption. Secondly, the biomarkers of PCP are not well-defined yet, and its active ingredients may act on different targets. Therefore, this study only describes the hypoglycemic effect of PCP at the level of the animal as a whole. In addition, whether the active ingredients of PCP exert hypoglycemic effects through the gut- pancreas-liver axis is a direction we would like to investigate deeply in the future.

## Conclusion

In summary, our results demonstrated that PCP could ameliorate hyperglycemia, hyperlipidemia, IR, and expression of inflammatory cytokines in *db/db* mice by modulating the gut microbiota, which manifested by decreasing the abundance of LPS- and BSH-producing bacteria while enriching SCFA-producing and anti-inflammatory bacteria. Our findings provide a new basis for the application of PCP in diabetic treatment.

## Data Availability

All data generated or analyzed during this study are included in this published article. The raw data are available from the corresponding author on reasonable request.

## References

[1] Practice C, American Diabetes Association Professional (2022). 2. Classification and diagnosis of diabetes: standards of medical care in diabetes-2022. Diabetes Care.

[2] Hui S, Liu Y, Chen M, Wang X, Lang H, Zhou M, Yi L, Mi M (2019). Capsaicin improves glucose tolerance and insulin sensitivity through modulation of the gut microbiota-bile Acid-FXR Axis in type 2 Diabetic db/db mice. Mol Nutr Food Res.

[3] Qi Y, Wang X (2023). The role of gut microbiota in high-fat-diet-induced diabetes: lessons from animal models and humans. Nutrients.

[4] Wang Y, Wang X, Xiao X, Yu S, Huang W, Rao B, Chen F (2023). A single strain of Lactobacillus (CGMCC 21661) exhibits stable glucose- and lipid-lowering effects by regulating gut microbiota. Nutrients.

[5] Gérard C, Vidal H (2019). Impact of gut microbiota on host Glycemic Control. Front Endocrinol (Lausanne).

[6] Silva YP, Bernardi A, Frozza RL (2020). The role of short-chain fatty acids from gut microbiota in gut-brain communication. Front Endocrinol (Lausanne).

[7] Fluitman KS, Wijdeveld M, Nieuwdorp M, Rg IJ (2018). Potential of butyrate to influence food intake in mice and men. Gut.

[8] Tilg H, Zmora N, Adolph TE, Elinav E (2020). The intestinal microbiota fuelling metabolic inflammation. Nat Rev Immunol.

[9] Zhao L, Xuan Z, Song W, Zhang S, Li Z, Song G, Zhu X, Xie H, Zheng S, Song P (2020). A novel role for farnesoid X receptor in the bile acid-mediated intestinal glucose homeostasis. J Cell Mol Med.

[10] Deutschmann K, Reich M, Klindt C, Dröge C, Spomer L, Häussinger D, Keitel V (2018). Bile acid receptors in the biliary tree: TGR5 in physiology and disease. Biochim Biophys Acta Mol Basis Dis.

[11] Mori H, Svegliati Baroni G, Marzioni M, Di Nicola F, Santori P, Maroni L, Abenavoli L, Scarpellini E (2022). Farnesoid X receptor, bile acid metabolism, and gut microbiota. Metabolites.

[12] Chen Y, Li T, Tan P, Shi H, Cheng Y, Cai T, Bai J, Du Y, Fu W (2022). Kaempferol from Penthorum chinense Pursh attenuates hepatic Ischemia/Reperfusion Injury by suppressing oxidative stress and inflammation through activation of the Nrf2/HO-1 signaling pathway. Front Pharmacol.

[13] Zhao X, Zhou M, Deng Y, Guo C, Liao L, He L, Peng C, Li Y (2022). Functional teas from Penthorum chinense Pursh alleviates Ethanol-Induced hepatic oxidative stress and autophagy impairment in zebrafish via modulating the AMPK/p62/Nrf2/mTOR Signaling Axis. Plant Foods Hum Nutr.

[14] Nabi F, Tao W, Ye R, Li Z, Lu Q, Shang Y, Hu Y, Fang J, Bhutto ZA, Liu J (2022). Penthorum Chinense Pursh Extract alleviates aflatoxin B1-Induced Liver Injury and oxidative stress through mitochondrial Pathways in Broilers. Front Vet Sci.

[15] Gandhi GR, Vasconcelos ABS, Wu DT, Li HB, Antony PJ, Li H, Geng F, Gurgel RQ, Narain N, Gan RY (2020). Citrus Flavonoids as promising phytochemicals targeting diabetes and related complications: a systematic review of in vitro and in vivo studies. Nutrients.

[16] Cao H, Ou J, Chen L, Zhang Y, Szkudelski T, Delmas D, Daglia M, Xiao J (2019). Dietary polyphenols and type 2 diabetes: human study and clinical trial. Crit Rev Food Sci Nutr.

[17] Jaja-Chimedza A, Zhang L, Wolff K, Graf BL, Kuhn P, Moskal K, Carmouche R, Newman S, Salbaum JM, Raskin I (2018). A dietary isothiocyanate-enriched moringa (Moringa oleifera) seed extract improves glucose tolerance in a high-fat-diet mouse model and modulates the gut microbiome. J Funct Foods.

[18] Sun Y, He L, Wang W, Wang T, Hua W, Li T, Wang L, Gao T, Chen F, Tang L (2021). Polyphenols from Penthorum chinense Pursh. Attenuates high glucose-induced vascular inflammation through directly interacting with Keap1 protein. J Ethnopharmacol.

[19] Huang D, Jiang Y, Chen W, Yao F, Huang G, Sun L (2015). Evaluation of hypoglycemic effects of polyphenols and extracts from Penthorum chinense. J Ethnopharmacol.

[20] Ke F, Xie P, Yang Y, Yan L, Guo A, Yang J, Zhang J, Liu L, Wang Q, Gao X (2021). Effects of Nisin, Cecropin, and Penthorum chinense Pursh on the intestinal microbiome of common carp (Cyprinus carpio). Front Nutr.

[21] Yin J, Ren W, Wei B, Huang H, Li M, Wu X, Wang A, Xiao Z, Shen J, Zhao Y, Du F, Ji H, Kaboli PJ, Ma Y, Zhang Z, Cho CH, Wang S, Wu X, Wang Y (2020). Characterization of chemical composition and prebiotic effect of a dietary medicinal plant Penthorum chinense Pursh. Food Chem.

[22] Li X, Zhao W, Xiao M, Yu L, Chen Q, Hu X, Zhao Y, Xiong L, Chen X, Wang X, Ba Y, Guo Q, Wu X (2022). Penthorum chinense Pursh. Extract attenuates non-alcholic fatty liver disease by regulating gut microbiota and bile acid metabolism in mice. J Ethnopharmacol.

[23] Ma Q, Li Y, Li P, Wang M, Wang J, Tang Z, Wang T, Luo L, Wang C, Wang T, Zhao B (2019). Research progress in the relationship between type 2 diabetes mellitus and intestinal flora. Biomed Pharmacother.

[24] Tao W, Zhu W, Nabi F, Li Z, Liu J (2023). Penthorum chinense Pursh compound flavonoids supplementation alleviates aflatoxin B1-induced liver injury via modulation of intestinal barrier and gut microbiota in broiler. Ecotoxicol Environ Saf.

[25] Wu R, Zhou L, Chen Y, Ding X, Liu Y, Tong B, Lv H, Meng X, Li J, Jian T, Chen J (2022). Sesquiterpene glycoside isolated from loquat leaf targets gut microbiota to prevent type 2 diabetes mellitus in db/db mice. Food Funct.

[26] Gurung M, Li Z, You H, Rodrigues R, Jump DB, Morgun A, Shulzhenko N (2020). Role of gut microbiota in type 2 diabetes pathophysiology. EBioMedicine.

[27] Leustean AM, Ciocoiu M, Sava A, Costea CF, Floria M, Tarniceriu CC, Tanase DM (2018). Implications of the Intestinal Microbiota in Diagnosing the Progression of Diabetes and the Presence of Cardiovascular Complications. J Diabetes Res.

[28] Zhao Q, Hou D, Fu Y, Xue Y, Guan X, Shen Q. (2021) Adzuki Bean alleviates obesity and insulin Resistance Induced by a High-Fat Diet and modulates gut microbiota in mice. Nutrients 13.https://www.ncbi.nlm.nih.gov/pubmed/34579118. 10.3390/nu13093240. 10.3390/nu13093240 PMC8466346 34579118

[29] Zhang Y, Zhou XA, Liu C, Shen Q, Wu Y (2022). Vitamin B6 inhibits high Glucose-Induced Islet β cell apoptosis by upregulating Autophagy. Metabolites..

[30] Kuo CS, Chen JS, Lin LY, Schmid-Schönbein GW, Chien S, Huang PH, Chen JW, Lin SJ (2020). Inhibition of serine protease activity protects against high Fat Diet-Induced inflammation and insulin resistance. Sci Rep.

[31] Du YC, Lai L, Zhang H, Zhong FR, Cheng HL, Qian BL, Tan P, Xia XM, Fu WG (2020). Kaempferol from Penthorum chinense Pursh suppresses HMGB1/TLR4/NF-κB signaling and NLRP3 inflammasome activation in acetaminophen-induced hepatotoxicity. Food Funct.

[32] Lin LM, Zhao LJ, Deng J, Xiong SH, Tang J, Li YM, Xia BH, Liao DF (2018). Enzymatic Extraction, Purification, and Characterization of Polysaccharides from Penthorum chinense Pursh: Natural Antioxidant and Anti-Inflammatory. Biomed Res Int.

[33] Guo WW, Wang X, Chen XQ, Ba YY, Zhang N, Xu RR, Zhao WW, Wu X (2018). Flavonones from Penthorum chinense ameliorate hepatic steatosis by activating the SIRT1/AMPK pathway in HepG2 cells. Int J Mol Sci.

[34] Gao JH, Wen SL, Tong H, Wang CH, Yang WJ, Tang SH, Yan ZP, Tai Y, Ye C, Liu R, Huang ZY, Tang YM, Yang JH, Tang CW (2016). Inhibition of cyclooxygenase-2 alleviates liver cirrhosis via improvement of the dysfunctional gut-liver axis in rats. Am J Physiol Gastrointest Liver Physiol.

[35] Svegliati-Baroni G, Patrício B, Lioci G, Macedo MP, Gastaldelli A (2020). Gut-pancreas-liver Axis as a target for treatment of NAFLD/NASH. Int J Mol Sci.

[36] Hernández MAG, Canfora EE, Jocken JWE, Blaak EE (2019). The short-chain fatty acid acetate in body weight control and insulin sensitivity. Nutrients.

[37] Yoshida H, Ishii M, Akagawa M (2019). Propionate suppresses hepatic gluconeogenesis via GPR43/AMPK signaling pathway. Arch Biochem Biophys.

[38] Zhang W, Xu JH, Yu T, Chen QK (2019). Effects of berberine and metformin on intestinal inflammation and gut microbiome composition in db/db mice. Biomed Pharmacother.

[39] Ghorbani Y, Schwenger KJP, Allard JP (2021). Manipulation of intestinal microbiome as potential treatment for insulin resistance and type 2 diabetes. Eur J Nutr.

[40] Xiao S, Fei N, Pang X, Shen J, Wang L, Zhang B, Zhang M, Zhang X, Zhang C, Li M, Sun L, Xue Z, Wang J, Feng J, Yan F, Zhao N, Liu J, Long W, Zhao L (2014). A gut microbiota-targeted dietary intervention for amelioration of chronic inflammation underlying metabolic syndrome. FEMS Microbiol Ecol.

[41] Liu X, Zhang Y, Li W, Zhang B, Yin J, Liuqi S, Wang J, Peng B, Wang S (2022). Fucoidan ameliorated Dextran Sulfate Sodium-Induced Ulcerative Colitis by modulating gut microbiota and bile acid metabolism. J Agric Food Chem.

[42] Barlow GM, Yu A, Mathur R (2015). Role of the gut microbiome in obesity and diabetes Mellitus. Nutr Clin Pract.

[43] Zhuang P, Li H, Jia W, Shou Q, Zhu Y, Mao L, Wang W, Wu F, Chen X, Wan X, Wu Y, Liu X, Li Y, Zhu F, He L, Chen J, Zhang Y, Jiao J (2021). Eicosapentaenoic and docosahexaenoic acids attenuate hyperglycemia through the microbiome-gut-organs axis in db/db mice. Microbiome.

[44] Weiss GA, Chassard C, Hennet T (2014). Selective proliferation of intestinal Barnesiella under fucosyllactose supplementation in mice. Br J Nutr.

[45] Ye J, Lee JW, Presley LL, Bent E, Wei B, Braun J, Schiller NL, Straus DS, Borneman J (2008). Bacteria and bacterial rRNA genes associated with the development of colitis in IL-10(-/-) mice. Inflamm Bowel Dis.

[46] Tao W, Yue X, Ye R, Nabi F, Shang Y, Zhu Z, Ahmed BZ, Liu J (2022). Hepatoprotective Effect of the Penthorum Chinense Pursh Extract against the CCl(4)-Induced Acute Liver Injury via NF-κB and p38-MAPK PATHWAYS in Dogs. Anim (Basel).

[47] Pereira FC, Wasmund K, Cobankovic I, Jehmlich N, Herbold CW, Lee KS, Sziranyi B, Vesely C, Decker T, Stocker R, Warth B, von Bergen M, Wagner M, Berry D (2020). Rational design of a microbial consortium of mucosal sugar utilizers reduces Clostridiodes difficile colonization. Nat Commun.

[48] Rodrigues RR, Gurung M, Li Z, García-Jaramillo M, Greer R, Gaulke C, Bauchinger F, You H, Pederson JW, Vasquez-Perez S, White KD, Frink B, Philmus B, Jump DB, Trinchieri G, Berry D, Sharpton TJ, Dzutsev A, Morgun A, Shulzhenko N (2021). Transkingdom interactions between Lactobacilli and hepatic mitochondria attenuate western diet-induced diabetes. Nat Commun.

[49] Zeng Z, Yuan Q, Yu R, Zhang J, Ma H, Chen S (2019). Ameliorative effects of probiotic lactobacillus paracasei NL41 on insulin sensitivity, oxidative stress, and Beta-cell function in a type 2 diabetes Mellitus Rat Model. Mol Nutr Food Res.

[50] Bordalo Tonucci L, Dos Santos KM, De Luces Fortes Ferreira CL, Ribeiro SM, De Oliveira LL, Martino HS (2017). Gut microbiota and probiotics: focus on diabetes mellitus. Crit Rev Food Sci Nutr.

[51] Karlsson FH, Tremaroli V, Nookaew I, Bergström G, Behre CJ, Fagerberg B, Nielsen J, Bäckhed F (2013). Gut metagenome in european women with normal, impaired and diabetic glucose control. Nature.

[52] Chávez-Talavera O, Tailleux A, Lefebvre P, Staels B (2017). Bile acid control of metabolism and inflammation in obesity, type 2 diabetes, Dyslipidemia, and nonalcoholic fatty liver disease. Gastroenterology.

[53] Kong B, Huang J, Zhu Y, Li G, Williams J, Shen S, Aleksunes LM, Richardson JR, Apte U, Rudnick DA, Guo GL (2014). Fibroblast growth factor 15 deficiency impairs liver regeneration in mice. Am J Physiol Gastrointest Liver Physiol.

[54] He X, Li W, Chen Y, Lei L, Li F, Zhao J, Zeng K, Ming J (2022). Dietary fiber of Tartary buckwheat bran modified by steam explosion alleviates hyperglycemia and modulates gut microbiota in db/db mice. Food Res Int.

[55] Liu Y, Dong M, Yang Z, Pan S (2016). Anti-diabetic effect of citrus pectin in diabetic rats and potential mechanism via PI3K/Akt signaling pathway. Int J Biol Macromol.

[56] Deledda A, Palmas V, Heidrich V, Fosci M, Lombardo M, Cambarau G, Lai A, Melis M, Loi E, Loviselli A, Manzin A, Velluzzi F (2022). Dynamics of Gut Microbiota and Clinical Variables after Ketogenic and Mediterranean Diets in Drug-Naïve Patients with Type 2 Diabetes Mellitus and Obesity. Metabolites.

[57] Tong X, Xu J, Lian F, Yu X, Zhao Y, Xu L, Zhang M, Zhao X, Shen J, Wu S, Pang X, Tian J, Zhang C, Zhou Q, Wang L, Pang B, Chen F, Peng Z, Wang J, Zhen Z, Fang C, Li M, Chen L, Zhao L (2018). Structural Alteration of Gut Microbiota during the Amelioration of Human Type 2 Diabetes with Hyperlipidemia by Metformin and a Traditional Chinese Herbal Formula: a Multicenter, Randomized. Open Label Clinical Trial. mBio.

[58] Sun RX, Huang WJ, Xiao Y, Wang DD, Mu GH, Nan H, Ni BR, Huang XQ, Wang HC, Liu YF, Fu Q, Zhao JX (2022). Shenlian (SL) Decoction, a Traditional Chinese Medicine Compound, May Ameliorate Blood Glucose via Mediating the Gut Microbiota in db/db Mice. J Diabetes Res.

